# Benchmarking engineered exchange interactions on NISQ hardware

**DOI:** 10.1038/s41598-026-53082-6

**Published:** 2026-05-25

**Authors:** Muhammad AbuGhanem

**Affiliations:** Faculty of Science, Ain Shams University, Cairo, 11566 Egypt

**Keywords:** Entanglement engineering, Engineered exchange interactions, Superconducting quantum computers, Quantum process tomography, NISQ era, *i*SWAP gate, $\sqrt{i\text {SWAP}}$ gate, Engineering, Physics

## Abstract

Engineered exchange interactions, realized through the *i*SWAP and $\sqrt{i\text {SWAP}}$ gates, play a fundamental role in entangling operations for quantum algorithms, simulation of spin-exchange dynamics, and optimized qubit connectivity. In this work, we present hardware-aware implementations of the *i*SWAP and $\sqrt{i\text {SWAP}}$ gates tailored to superconducting quantum processors, along with comprehensive characterization using both quantum process tomography (QPT) and direct state measurements (DSM). QPT results show process fidelities of 97.32% (*i*SWAP) and 98.02% ($\sqrt{i\text {SWAP}}$) on quantum simulator, decreasing to 89.72% and 87.65% on quantum hardware, respectively. DSM on the $|00\rangle$ input state reveals that the *i*SWAP implementation achieves higher state preservation fidelity on hardware (93.53% vs. 92.44% for $\sqrt{i\text {SWAP}}$) but shows higher measured $|11\rangle$ population (2.26% vs. 0.38%). These results establish a benchmark for anisotropic exchange gates on noisy intermediate-scale quantum (NISQ) hardware and provide quantitative performance data to inform gate selection for quantum circuit design in the NISQ era.

## Introduction

Quantum computers represent a paradigm shift in computing^1^, leveraging the principles of quantum mechanics—such as superposition and entanglement^2,3^—to solve problems intractable for classical computers^4–6^. This potential spans diverse fields^7^, from drug discovery^8^ and materials science^9^ to cryptography^10^ and financial modeling^11^. The realization of this promise, however, hinges on the ability to construct and control large-scale, fault-tolerant quantum processors^12,13^.

The pursuit of practical quantum computation^7,14,15^ relies on the precise implementation and characterization of high-fidelity entangling gates^15–17^. While the controlled-NOT (CNOT) and controlled-phase (CZ) gates are canonical choices for universal quantum computation^15,16,18,19^, alternative entangling interactions offer distinct advantages for specific applications, such as quantum simulation and efficient qubit routing^4,20,21^. Among these, anisotropic exchange interactions–mathematically realized through the *i*SWAP and $\sqrt{i\text {SWAP}}$ gates^16^—provide a powerful family of operations that naturally emerge in various physical platforms ^22–24^, including superconducting circuits^25^.

The *i*SWAP gate^16^, which coherently exchanges excitations between two qubits with a phase factor, and its square-root variant, $\sqrt{i\text {SWAP}}$^16^, which serves as a perfect entangler, are particularly valuable for simulating quantum magnetic systems^26^ and optimizing compilation in architectures with limited connectivity^27–30^. In IBM’s superconducting quantum processors^1^, these gates are not native but are compiled into the native gate set^31–33^, making their performance susceptible to the complex noise environment of NISQ (noisy intermediate-scale quantum) devices^17^. To mitigate these effects, we develop hardware-aware decompositions tailored to the specific native gate set, calibration constraints, and coupling map of the target device.

Quantum process tomography (QPT) is a powerful technique for fully characterizing a quantum operation^39–41^, providing a complete description of the implemented quantum channel. By reconstructing the Choi matrix, QPT yields not only the process fidelity–a single-number benchmark–but also reveals the underlying error mechanisms, offering valuable diagnostic insights^41^.

This work presents hardware-aware implementations and comprehensive experimental characterization of the *i*SWAP and $\sqrt{i\text {SWAP}}$ gates on IBM’s superconducting quantum hardware^7^. Employing a dual-methodology approach combining full QPT with DSM^39–41^, our analysis reveals a $\sim 9-10\%$ process fidelity reduction attributable to device noise, as well as differences in measured output populations when applying the gates to the $|00\rangle$ input state. These quantitative results provide a benchmark for evaluating gate performance on NISQ hardware. The protocols and implementations presented here are applicable to several near-term quantum applications, including simulating spin-exchange dynamics in magnetic systems^26^, optimizing qubit routing in connectivity-limited architectures^20,30^, and compiling efficient entangling operations for variational quantum algorithms^42^. Furthermore, we provide a detailed analysis of hardware metrics–from relaxation times to readout errors–shedding light on the practical viability of anisotropic exchange gates for quantum algorithms in the NISQ era^12,42^.

Recent progress in pulse-level control–including steep-edge pulse generation^34^ and adjustable pulse-current techniques^35^–offers hardware insights relevant to timing-aware gate implementation. Entanglement-sensitive measurement and visualization^36^ highlight the importance of detailed experimental characterization beyond single-fidelity metrics. More broadly, quantum-inspired optimization strategies^37,38^ demonstrate the growing intersection of classical optimization and quantum concepts, though their direct application to quantum circuit compilation remains an open direction.

The remainder of this paper is organized as follows. Section Methods describes the experimental setup, including the compilation of target gates into native hardware operations in Sect. Hardware-aware implementation of the *i*SWAP and $\sqrt{i\text {SWAP}}$ gates, DSM Protocol in Sect. Direct state measurement protocol, and the QPT technique in Sect. Quantum process tomography. The core results of our study are presented in Sect.  Results and discussion, where we present DSM results in Sect. Direct state measurements, compare simulated and experimental process fidelities in Sect. Quantum process fidelities, analyze the underlying error mechanisms, and discuss device comparative performance and quantum hardware analysis in Sect. Comparative performance and hardware analysis. Finally, Sect.  Conclusion provides concluding remarks and suggests avenues for future work.

## Methods

### Hardware-aware implementation of the *i*SWAP and $\sqrt{i\text {SWAP}}$ gates

**Fig. 1:**
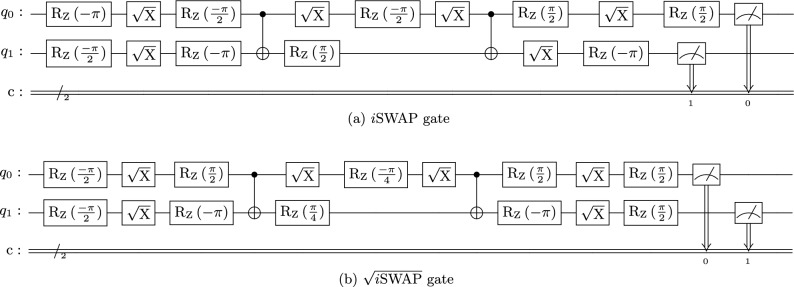
Hardware-aware quantum circuit implementations of engineered exchange interactions ($i\textrm{SWAP}$ and $\sqrt{i\textrm{SWAP}}$). (**a**) Decomposition of the $i\textrm{SWAP}$ gate into the native gate set $\{\texttt {CNOT}, \texttt {RZ}, \texttt {SX}\}$ using two CNOT gates interleaved with single-qubit $R_Z$ and $\textrm{SX}$ ($\sqrt{X}$) rotations. The matrix definitions are $$\textrm{SX} = \begin{pmatrix} \frac{1}{2}(1+i) & \frac{1}{2}(1-i) \\ \frac{1}{2}(1-i) & \frac{1}{2}(1+i) \end{pmatrix}$$ and $$R_Z(\gamma ) = \begin{pmatrix} e^{-i\gamma /2} & 0 \\ 0 & e^{i\gamma /2} \end{pmatrix}$$. (**b**) Decomposition of the $\sqrt{i\textrm{SWAP}}$ gate using the same native gate set, also requiring only two CNOT gates. Both compilations minimize two-qubit interactions to reduce error accumulation on NISQ hardware.

We developed hardware-aware implementations of the *i*SWAP and $\sqrt{i\text {SWAP}}$ gates compiled into quantum circuits comprising the native gate set, $\{\texttt {CNOT}, \texttt {RZ}, \texttt {SX}\}$, targeting the specific coupling map and qubit properties of IBM’s superconducting *Falcon* architecture, specifically the ibm_perth quantum computer. Figure 1a presents the quantum circuit that implements the *i*SWAP gate using a hardware-native gate set. The corresponding decomposition for the $\sqrt{i\text {SWAP}}$ gate is shown in Fig. 1b. Both circuits are engineered for realistic NISQ-era hardware, utilizing a minimal number of two-qubit interactions (only two CNOT gates) interleaved with single-qubit rotations, thereby minimizing latency and error accumulation.

### Direct state measurement protocol

To assess the output state distribution and error patterns of each gate when applied to $|00\rangle$, we performed DSM by preparing the $|00\rangle$ input state, applying either the *i*SWAP or $\sqrt{i\text {SWAP}}$ gate, and measuring in the computational basis. For each gate, we executed 7,168 shots on both the qasm_simulator (for ideal reference) and the ibm_perth quantum computer. The output distribution across the four computational basis states ($|00\rangle$, $|01\rangle$, $|10\rangle$, $|11\rangle$) was recorded, providing insights into:

- State preservation fidelity ($P_{00}$)
- Single-qubit excitation errors ($P_{01}$, $P_{10}$)
- Two-qubit error events ($P_{11}$)

Because both gates act trivially on the $|00\rangle$ state, any measured population in $|01\rangle$, $|10\rangle$, or $|11\rangle$ directly reflects gate imperfections, hardware noise, or readout errors. This protocol complements QPT by providing additional characterization of output state distributions under identical initial conditions.

### Quantum process tomography

We employed standard full QPT^39,40,44^ to fully characterize the implemented gates. Each QPT experiment was executed with 4,000, providing sufficient statistical accuracy for the reconstruction. The resulting data was subsequently processed to reconstruct the physical Choi matrix $\chi$ that best describes the experimental quantum process^41^. The performance of the gate was quantified by calculating the process fidelity ($\mathcal {F}_{\text {process}}$)^45,46^, which measures the overlap between the experimentally reconstructed process ($\chi _{\text {exp}}$) and the ideal target process ($\chi _{\text {ideal}}$).

All experiments were conducted on IBM Quantum’s ibm_perth quantum computer, a 7-qubit *Falcon* r5.11H system with a quantum volume of 32^47^, using qubits 0 and 1. The device’s key performance metrics, including relaxation times ($T_1$), dephasing times ($T_2$), and readout errors ($\delta _r$), are detailed in Tables 1 and 2. These metrics were recorded concurrently with the full QPT experiments to provide a snapshot of the device’s performance.

**Fig. 2:**
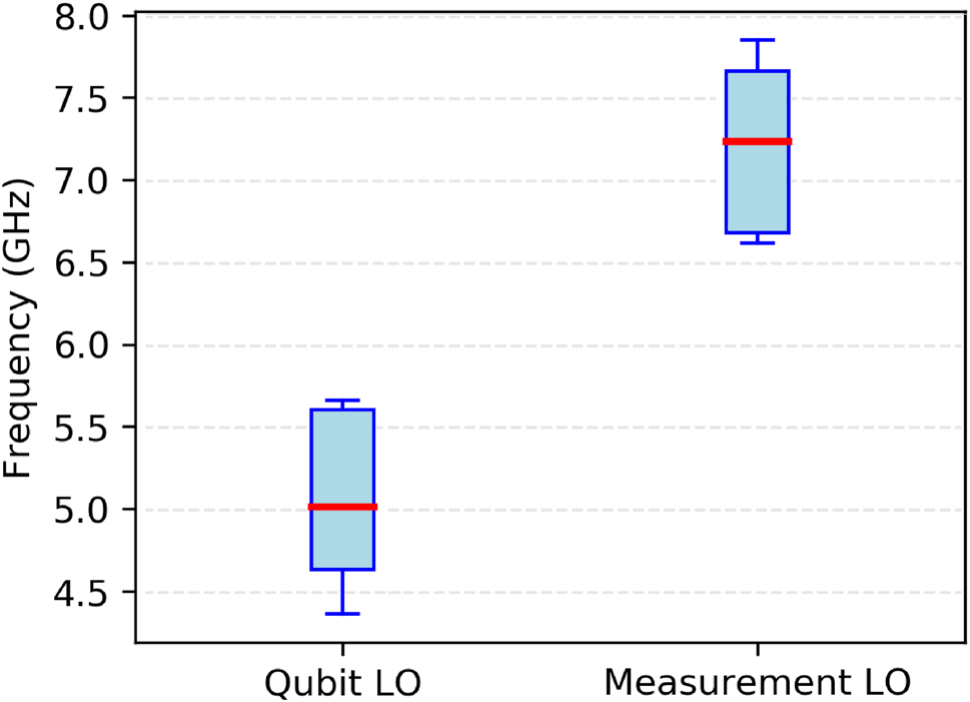
Distribution of local oscillator frequencies for qubit control and readout. Box plot comparing the operational frequency ranges (in GHz) of the local oscillators (LOs) used for qubit drive (Qubit LO) and measurement readout (Measurement LO) across the seven-qubit processor.

As shown in Fig. 2, the local oscillator frequencies for qubit drive and measurement are well separated into two distinct frequency bands, minimizing the risk of control-readout interference^48,49^. The qubit LOs operate in the $\sim 4-5$ GHz range (approximately 4.36–5.66 GHz), corresponding to the transition frequencies of the transmon qubits^25^. While the measurement LOs are systematically up-converted to the $\sim 6-7$ GHz range (approximately 6.62–7.85 GHz), aligning with the frequencies of the readout resonators and leveraging the dispersive shift for state-selective measurement. This frequency separation is a critical hardware design feature that ensures operational fidelity^32^.

**Table 1:** Qubit-specific performance characteristics recorded during the QPT experiments of the *i*SWAP gate. Metrics include relaxation time ($T_1$), dephasing time ($T_2$), frequency ($\Omega /2\pi$), anharmonicity ($\Lambda$), readout error ($\delta _r$), conditional measurement probabilities (*P*(0|1), *P*(1|0)), and readout duration ($\varepsilon _r$).

Qubit	T$_1$ ($\mu$s)	T$_2$ ($\mu$s)	$\Omega /2\pi$ (GHz)	$\Lambda$ (GHz)	$\delta _r$	*P*(0|1)	*P*(1|0)	$\varepsilon _r$ (ns)
0	112.217	89.958	5.15756	-0.34152	0.0281	0.0292	0.0270	721.78
1	143.980	54.158	5.03355	-0.34437	0.0304	0.0320	0.0288	721.78
2	214.031	95.996	4.86266	-0.34727	0.0338	0.0272	0.0404	721.78
3	168.649	227.967	5.12510	-0.34044	0.0161	0.0186	0.0136	721.78
4	169.539	132.514	5.15921	-0.33337	0.0293	0.0286	0.0300	721.78
5	148.766	142.089	4.97861	-0.34602	0.0300	0.0344	0.0256	721.78
6	188.841	231.382	5.15664	-0.34045	0.0114	0.0126	0.0102	721.78

**Table 2:** Calibration data from the IBM Quantum device during the $\sqrt{i\text {SWAP}}$ QPT experiments, providing context for the error sources contributing to the observed experimental fidelity.

Qubit	T$_1$ ($\mu$s)	T$_2$ ($\mu$s)	$\Omega /2\pi$ (GHz)	$\Lambda$ (GHz)	$\delta _r$	*P*(0|1)	*P*(1|0)	$\varepsilon _r$ (ns)
0	87.651	89.958	5.15756	-0.34152	0.0281	0.0292	0.0270	721.78
1	193.942	54.158	5.03355	-0.34437	0.0304	0.0320	0.0288	721.78
2	96.764	95.996	4.86266	-0.34727	0.0338	0.0272	0.0404	721.78
3	201.576	227.967	5.12510	-0.34044	0.0161	0.0186	0.0136	721.78
4	154.494	132.514	5.15921	-0.33337	0.0293	0.0286	0.0300	721.78
5	128.461	142.089	4.97861	-0.34602	0.0300	0.0344	0.0256	721.78
6	222.168	231.382	5.15664	-0.34045	0.0114	0.0126	0.0102	721.78

## Results and discussion

### Direct state measurements

Experimental characterization of the *i*SWAP-family gates reveals distinct output state distributions when applied to the $|00\rangle$ input state. The results are summarized in Table 3.

For the *i*SWAP gate, execution on the noiseless simulator yielded ideal performance with all 7,168 shots returning the $|00\rangle$ state ($P_{00}^{\text {sim}} = 100\%$). On quantum hardware, the gate achieved $P_{00}^{\text {hw}} = 93.53\%$, with the 6.47% infidelity distributed across erroneous states. The measured $|11\rangle$ population was 2.26%. The $\sqrt{i\text {SWAP}}$ gate showed a different population distribution. While simulation maintained perfect results ($P_{00}^{\text {sim}} = 100\%$), hardware execution achieved $P_{00}^{\text {hw}} = 92.44\%$. The measured $|11\rangle$ population was substantially lower at 0.38%, while single-excitation populations in $|01\rangle$ (3.10%) and $|10\rangle$ (4.09%) were higher than for the *i*SWAP gate.

**Fig. 3:**
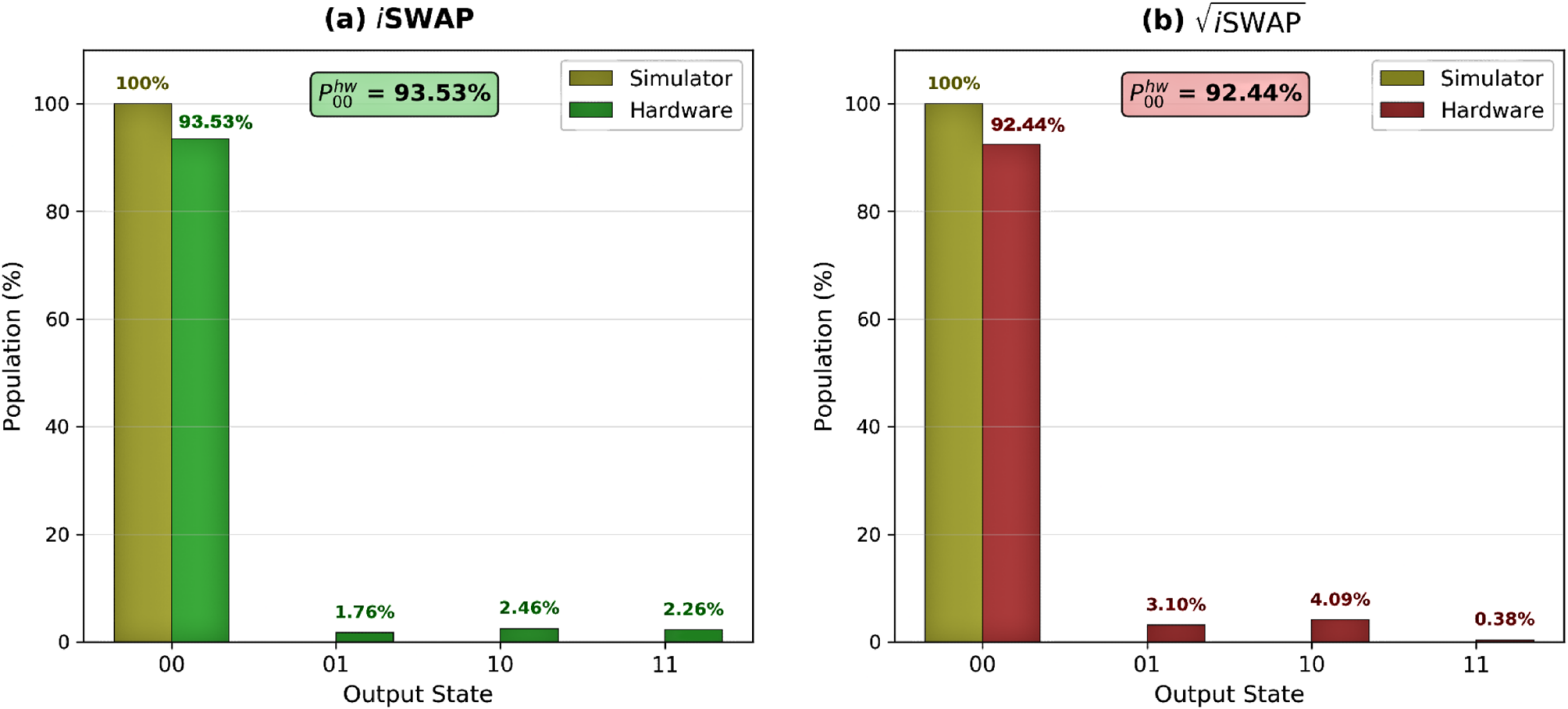
Output state populations for (**a**) *i*SWAP and (**b**) $\sqrt{i\textrm{SWAP}}$ gates applied to the $|00\rangle$ input state.

Because both gates act trivially on $|00\rangle$, any population in $|01\rangle$, $|10\rangle$, or $|11\rangle$ arises from hardware noise, gate imperfections, or readout errors. The observed differences in output distributions reflect the distinct error sensitivities of the two compiled implementations under the same device noise environment. Figure 3 shows the output state distributions for both gates. While the DSM protocol presented here probes only the $|00\rangle$ state, the complete process characterization is provided by QPT (Sect. Quantum process fidelities).

**Table 3:** Measured output state distributions for *i*SWAP and $\sqrt{i\text {SWAP}}$ gates applied to $|00\rangle$ input (7,168 shots).

Gate	Platform	$\mathbf {P_{00}}$ (%)	$\mathbf {P_{01}}$ (%)	$\mathbf {P_{10}}$ (%)	$\mathbf {P_{11}}$ (%)
*i*SWAP	Simulator	100.00	0.00	0.00	0.00
*i*SWAP	Hardware	93.53	1.76	2.46	2.26
$\sqrt{i\text {SWAP}}$	Simulator	100.00	0.00	0.00	0.00
$\sqrt{i\text {SWAP}}$	Hardware	92.44	3.10	4.09	0.38

**Fig. 4:**
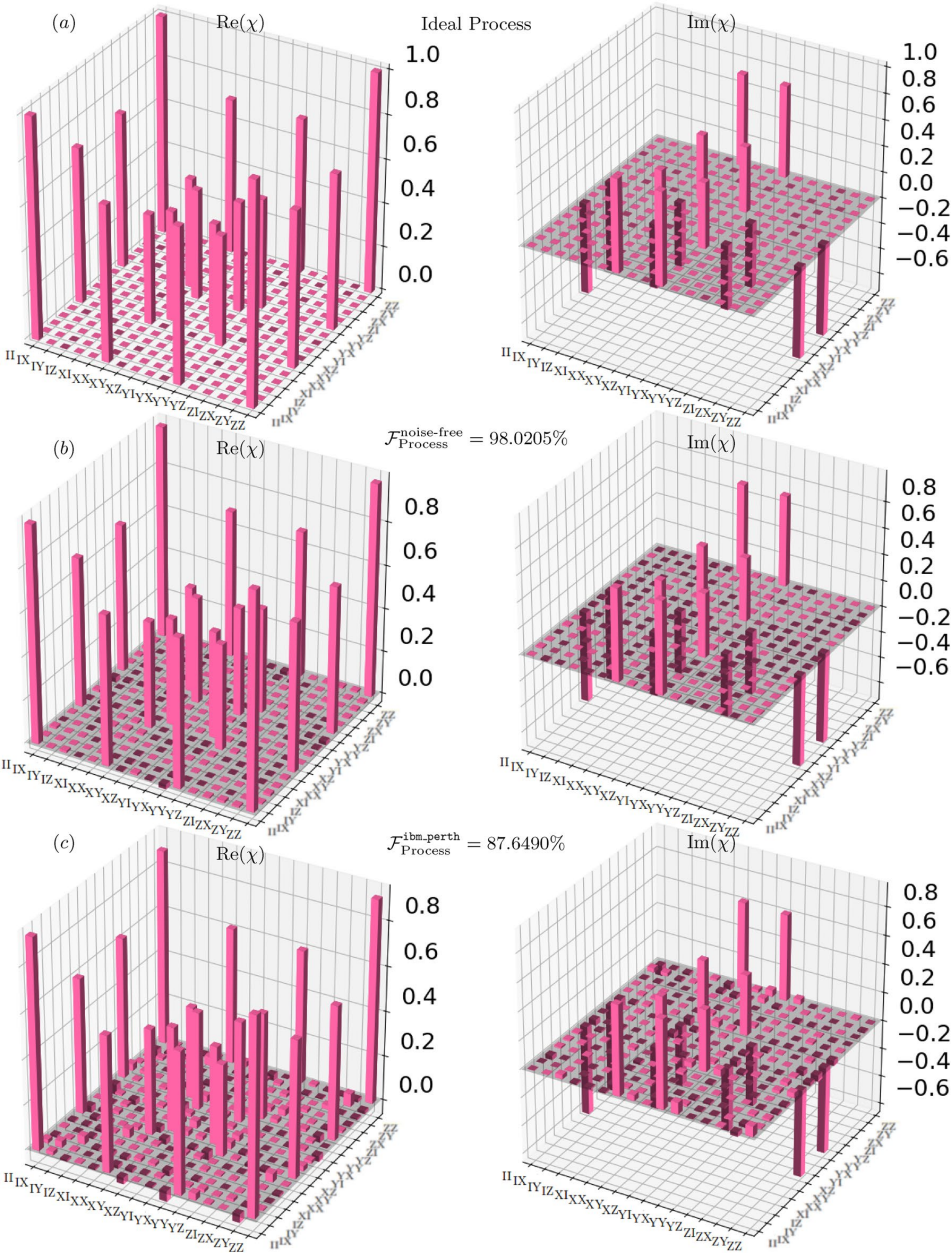
Experimental characterization of the $\sqrt{i\text {SWAP}}$ gate via QPT. (**a**) The ideal process matrix. (**b**) The noise-free simulation result ($\mathcal {F}{\text {sim}} = 98.02\%$). (**c**) The process reconstructed from data acquired on the ibm_perth quantum computer ($\mathcal {F}{\text {exp}} = 87.65\%$).

**Fig. 5:**
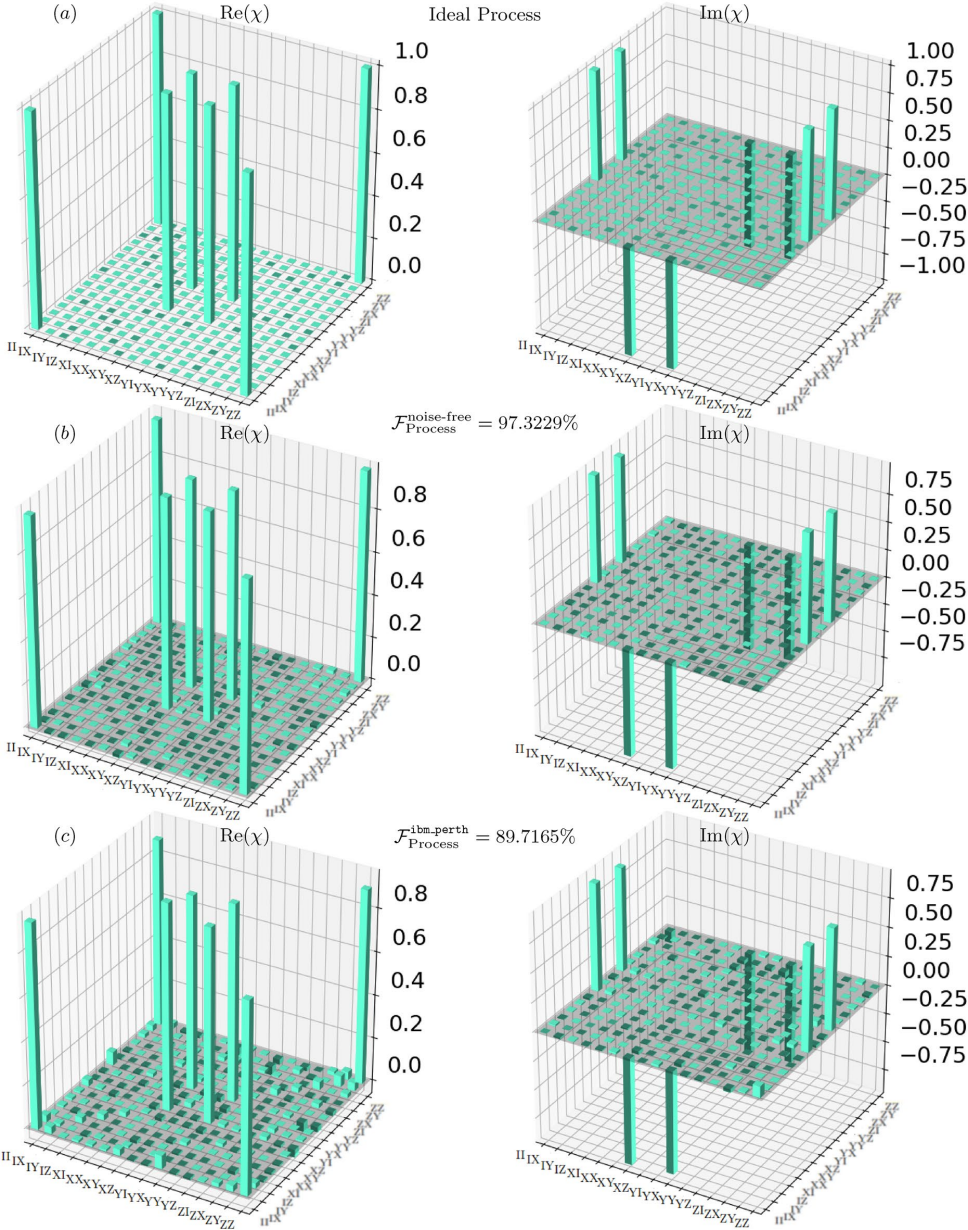
Quantum process tomography of the *i*SWAP gate. (**a**) The ideal Choi matrix representing the target unitary. (**b**) The reconstructed process from a noiseless simulation, achieving a fidelity of $\mathcal {F}{\text {sim}} = 97.32\%$. (**c**) The experimental implementation on the ibm_perth, yielding a fidelity of $\mathcal {F}{\text {exp}} = 89.72\%$. The real (Re$(\chi )$) and imaginary (Im$(\chi )$) components of the Choi matrix $\chi$ are shown for each case.

### Quantum process fidelities

The experimental quantum process fidelities from our full QPT experiments are summarized in Table 4 (including a comparison with the native CNOT gate) and visually presented in Figs. 4 and 5. The observed fidelities reflect the effectiveness of our hardware-aware implementations.

Execution on IBM Quantum’s qasm_simulator established a theoretical performance upper bound^50^, yielding high fidelities of 97.32% for the *i*SWAP gate and 98.02% for the $\sqrt{i\text {SWAP}}$ gate. While the simulator is noiseless (no decoherence or gate errors), the finite number of shots introduces statistical fluctuations in the measurement outcomes. As a result, the reconstructed process fidelity deviates from unity even for ideal gates, following the fundamental scaling law for sampling noise^51^. The statistical uncertainty scales as $\mathcal {O}(1/\sqrt{N_{\text {shots}}})$; with 4,000 shots, the reconstructed fidelity is below unity and converges to unity as $N_{\text {shots}}$ increases. Thus, the residual infidelity of $\sim 2-3\%$ arises from finite-sampling shot noise and is distinct from hardware noise^51,52^.

Experimental execution on the quantum hardware revealed a significant fidelity reduction due to device noise. The *i*SWAP gate achieved a process fidelity^45,46^ of 89.72%, while the $\sqrt{i\text {SWAP}}$ gate achieved 87.65%. This $\sim 9-10\%$ drop is a direct consequence of decoherence, imperfect gate calibration, and state preparation and measurement (SPAM) errors endemic to NISQ-era devices^53,54^. Visual inspection of the reconstructed Choi matrices in Figs. 4 and 5 provide a qualitative assessment. The experimental matrices (subfigures (c)) show clear deviations from the ideal ones (a), particularly in the off-diagonal elements which correspond to coherent errors and energy loss^53^.

**Table 4:** Characterization of *i*SWAP, $\sqrt{i\text {SWAP}}$, and CNOT gates showing QPT fidelities ($\mathcal {F}_p$) and output state populations ($P_{00}$) for the $|00\rangle$ input. The $\sqrt{i\text {SWAP}}$ gate shows the largest hardware fidelity drop ($\Delta \mathcal {F}_p = 10.37\%$), while the CNOT gate exhibits the smallest ($4.87\%$). All three gates show comparable $P_{00}$ degradation on hardware ($\sim 6.5-7.6\%$).

Gate	Process fidelity $\mathcal {F}_p$		$\Delta \mathcal {F}_p$	State preparation $P_{00}$		$\Delta P_{00}$
	Simulator (%)	Hardware (%)	(HW Drop) (%)	Simulator (%)	Hardware (%)	(HW Drop) (%)
*i*SWAP (This study)	97.32	89.72	7.60	100.00	93.53	6.47
$\sqrt{i\text {SWAP}}$ (This study)	98.02	87.65	10.37	100.00	92.44	7.56
CNOT ^55^	97.89	93.02	4.87	100.00	92.40	7.60

### Comparative performance and hardware analysis

**Fig. 6:**
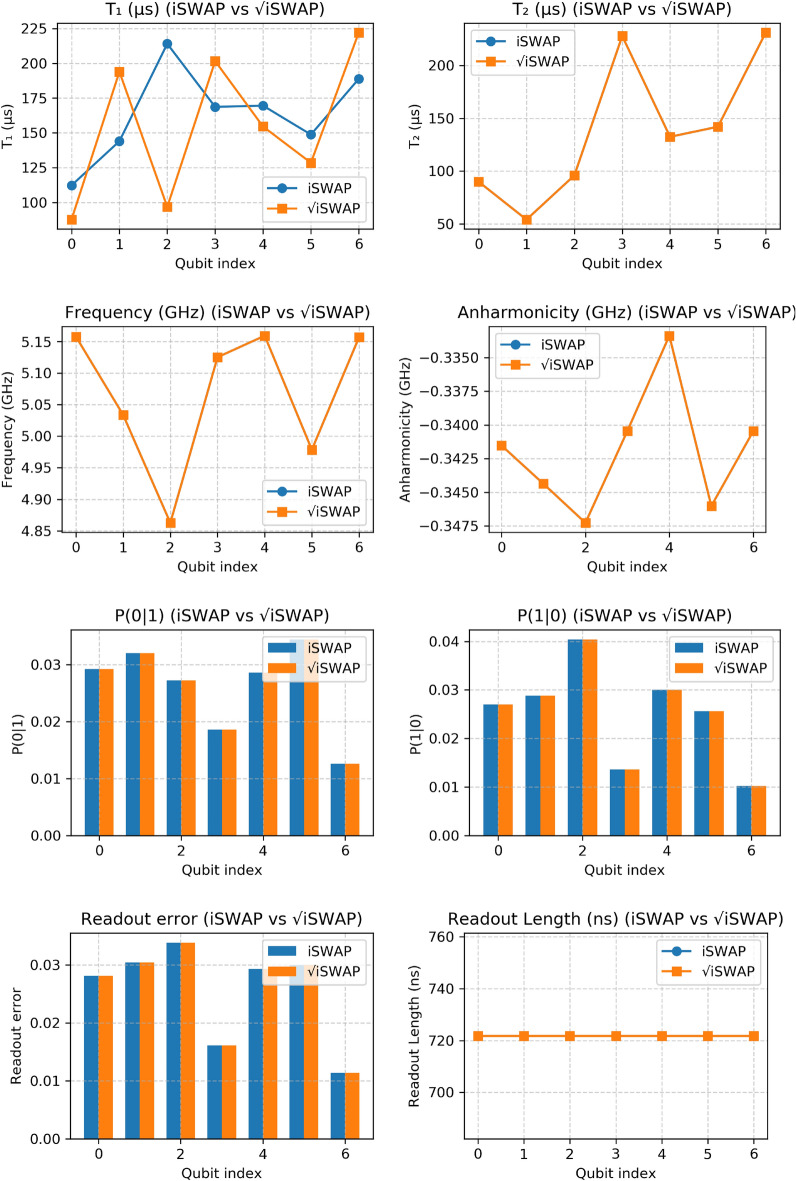
Device performance metrics during QPT execution for the *i*SWAP and $\sqrt{i\text {SWAP}}$ gates. Key parameters—including relaxation time ($T_1$), dephasing time ($T_2$), qubit frequency ($\Omega /2\pi$), anharmonicity ($\Lambda$), readout error ($\delta _r$), state-assignment error (*P*(0|1), *P*(1|0)), and readout length—are compared across the seven qubits.

A comprehensive analysis of the hardware performance during these experiments is provided in Fig. 6. The plots compare key qubit metrics–$T_1$, $T_2$, frequency, anharmonicity, and readout errors–for both gates. Our benchmarking experiments used qubits 0 and 1 (see Tables 1 and 2). Considering first these two qubits, we observe that qubit 1 exhibits a substantially shorter $T_2$ (54.2 $\mu$s) than qubit 0 (90.0 $\mu$s), indicating higher dephasing sensitivity in qubit 1. More broadly, the full 7-qubit data in Fig. 6 reveals considerable qubit-to-qubit variability, a hallmark of current superconducting processors.

Several observations can be made from these plots. For the $T_1$ metric, significant variation is evident across qubits and between experimental runs (e.g., qubit 0 $T_1$ decreased from 112.2 $\mu$s to 87.7 $\mu$s), highlighting the stochastic nature of decoherence and device drift in NISQ processors^17^. In contrast, $T_2$ values remained stable across both experiments (see Tables 1 and 2). The qubit frequency remains stable across both experimental runs, as expected for fixed-frequency transmon qubits^25^.

## Conclusion

In this work, we have provided hardware-aware implementations and comprehensive experimental characterization of engineered exchange interactions, realized through the *i*SWAP and $\sqrt{i\text {SWAP}}$ gates, on a superconducting quantum processor. By employing a dual-methodology approach combining full quantum process tomography with direct state measurements, we have quantified their performance and established a multi-faceted benchmark of direct relevance for NISQ-era computation. The hardware-aware decompositions developed for this work minimize circuit depth by utilizing only two CNOT gates, demonstrating the effectiveness of targeted compilation for NISQ-era devices.

Our results reveal a nuanced performance landscape on current hardware. While quantum simulations yield high process fidelities exceeding 97%, execution on a superconducting quantum processor reveals a significant fidelity reduction to approximately 90% for *i*SWAP and $\sqrt{i\text {SWAP}}$. For context, the native CNOT gate achieves a fidelity of 93%, indicating that our implementations are competitive despite the additional circuit complexity. Direct state measurements provide complementary insights: when applied to the $|00\rangle$ input state, the *i*SWAP implementation shows higher measured $P_{00}$ (93.53% vs. 92.44%) but also a higher measured $|11\rangle$ population (2.26% vs. 0.38% for $\sqrt{i\text {SWAP}}$). The $\sim 10$ percentage point process fidelity gap reflects the cumulative impact of device noise, while the observed differences in output state distributions highlight the distinct error sensitivities of the two compiled implementations under the same noise environment.

Looking forward, this comprehensive benchmarking establishes a foundation for several future directions—optimizing gate selection based on specific error tolerance requirements, developing error mitigation strategies tailored to dominant error channels, and guiding the design of exchange-based quantum simulations. Extensions to other hardware platforms and integration of these gates into full quantum algorithms represent important next steps. As superconducting hardware continues to evolve, the rigorous multi-metric characterization demonstrated here remains essential for mapping progress toward fault-tolerant quantum computation.

## Data Availability

The datasets generated during and/or analyzed during this study are included within this article.
